# The New Era of Transplant Oncology: Liver Transplantation for Nonresectable Colorectal Cancer Liver Metastases

**DOI:** 10.1155/2018/9531925

**Published:** 2018-01-10

**Authors:** Andre Gorgen, Hala Muaddi, Wei Zhang, Ian McGilvray, Steven Gallinger, Gonzalo Sapisochin

**Affiliations:** ^1^Multi-Organ Transplant Unit, Department of Surgery, Toronto General Hospital, University Health Network, University of Toronto, Toronto, ON, Canada; ^2^Division of General Surgery, Department of Surgery, Toronto General Hospital, University Health Network, University of Toronto, Toronto, ON, Canada

## Abstract

Colorectal cancer (CRC) is the third most incident cancer worldwide. Most of CRC patients will develop distant metastases, mainly to the liver, and liver resection is the only potential chance for cure. On the other hand, only a small proportion of patients with hepatic CRC metastasis are candidates for upfront liver resection. Liver transplantation (LT) is an attractive option for patients with nonresectable CRC liver metastases (NRCLM) without extrahepatic involvement. Initial experiences with LT for NRCLM achieved very poor outcomes, with a 5-year overall survival (OS) lower than 20%. However, these initial studies did not have a standardized patient selection or neoadjuvant or adjuvant therapies. With recent advances in the surgical and medical oncology fields, the landscape has changed. Recent studies from Norway have shown an encouraging 5-year OS of 50% when transplanting patients with NRCLM. Nevertheless, the main concern when expanding the indications for LT is organ shortage. To manage this organ shortage, strategies utilizing live donor liver transplantation are gaining favor. A few ongoing trials are assessing the impact of LT in NRCLM patient survival. Therefore, the aim of this paper is to review the current status of LT for NRCLM.

## 1. Background

Colorectal cancer (CRC) is the third most common diagnosed cancer and the fourth cause of cancer-related mortality worldwide [1]. In 2012, there were over 1.3 million new cases diagnosed and approximately 700,000 related deaths worldwide [1]. In Canada, CRC accounts for 13% of all cancers. In 2017, CRC has a projected incidence of 80 new cases per 100.000 people and 9,400 deaths related to this cancer [2]. The CRC incidence is higher in developed countries and its rising among people younger than 55 years old [3, 4]. Approximately 15–25% of CRC patients will have distant metastases at the time of primary diagnosis, mainly to the liver [5]. Another 18%–25% patients will develop distant metastases within 5 years from the first diagnosis [6]. Over the past decade, the survival of patients with metastatic CRC has improved substantially [7]. The 2-year overall survival (OS) for stage IV CRC increased from 21% in the 1990s to 35% in the 2010s [8]. These improved outcomes are mainly a consequence of improved efficacy of systemic therapies and an increase in the number of patients undergoing surgical treatment for CRC metastasis [7, 8].

Surgical resection combined with neoadjuvant and adjuvant chemotherapy is the standard curative treatment for colorectal liver metastases (CRLM) [9, 10]. Several studies have shown 5-year OS rates of 47%–60% after hepatectomy for colorectal metastases [11–13]. However, recurrence occurs in 40%–75% of patients after liver resection [14–16]. Of these recurrences, 50% occur in the liver [15–17]. Repeated hepatectomy after hepatic recurrence has proven to be feasible and improves survival [18]. In contrast, the prognosis after recurrence of nonresectable CRC is dismal and the 5-year survival is less than 10% with palliative chemotherapy [19].

Liver resection is only feasible in 20–40% of patients with CRLM [20]. A common issue that precludes liver resection in these patients is the insufficient liver remnant volume due to large burden of metastatic disease [21]. Given that liver resection in combination with chemotherapy is the best treatment option for these patients and only a minority are eligible for this treatment, liver transplantation (LT) is an attractive option. LT would offer an R0 resection with the widest possible margin. Despite this being a potential choice for patients with this disease, the disparity between recipients in need of a LT and the number of available grafts continues to grow. Wait-list mortality is known to be ~25% depending on the jurisdiction [22, 23]. Therefore expanding the current indications for LT represents an ethical dilemma [24]. This review describes the current status of LT for NRCLM, focusing on the existing experience described in literature before and after 2000. Furthermore, we will discuss the potential role of living-donor liver transplantation for the management of NRCLM given the organ shortage and the future benefit of novel therapies for NRCLM.

## 2. Liver Transplantation for Malignancy

Several advances have been made in the field of LT over the past decades [25]. The establishment of patient selection criteria and guidelines for the management of hepatocellular carcinoma (HCC) together with the technical advances in the field of LT has dramatically improved patient outcomes [26]. Furthermore, the enhanced knowledge of HCC staging and biology and the adoption of the MELD system with exception points for HCC were also responsible for the improvement in the 5-year survival rates [28]. Today, patients transplanted for HCC achieve a 5-year OS ~75% representing the best treatment option for these patients [25]. The proportion of patients with HCC awaiting a LT in most transplant centers is ~40% representing one of the most frequent indications for LT [27, 28]. The experience achieved with LT for HCC has developed the foundation to expand the indication of LT to other malignancies under specific selection criteria. Cholangiocarcinoma, he patoblastoma, and neuroendocrine tumor liver metastases are currently accepted indications for LT in many jurisdictions with satisfactory survival rates [29–31]. These recent advances associated with the development of multidisciplinary teams in most academic institutions have opened a new era in the field of* Transplant Oncology* [32].

## 3. Results of LT for Nonresectable CRC Liver Metastases before 2000

Prior to 2000, the largest single-center experience with LT for NRCLM was performed at the Medical University of Vienna. This group reported 25 patients transplanted from 1982 to 1994 [33, 34]. In this series, the 1-, 3-, and 5-year OS was 76%, 32%, and 12%, respectively [35] with a 30-day mortality of ~30% [33]. One of the most interesting findings in the Austrian experience is that the indication of LT was restricted to patients with negative lymph nodes on the primary tumor specimen pretty early in their patient accrual, and this improved the outcomes after LT [36]. Following up with this observation, the same group conducted a recent retrospective analysis to assess the presence of micrometastases in the histologically negative lymph nodes of the primary colon cancer in their LT cohort [36]. They found that 15 of 21 patients that initially were classified as lymph node negative had in fact micrometastases. The median survival of patients without lymph node micrometastases that underwent a LT was 118 months whereas patients with lymph node micrometastases survived a median of 28 months (*P* = 0.01) [36].

In 1991, a heterogeneous cohort of 637 patients with primary and metastatic malignances to the liver that underwent LT at the University of Cincinnati was published [37]. The 30-day mortality in this series was 11%. In this cohort, 41 patients underwent LT for secondary malignances to the liver of which eight were transplanted for NRCLM and two underwent LT due to chemotherapy-related liver failure. The recurrence rate among CRC patients was 70%. The 2- and 5-year survival of patients who underwent LT for all types of metastatic malignances to the liver was 38% and 21%, respectively. Unfortunately, the authors did not provide specific survival for NRCLM patients [37] and therefore meaningful conclusions from this study are difficult to obtain.

Data from the European Liver Transplant Registry presented 58 cases of LT for NRCLM performed between 1977 and 1995. The reported 1-, 3-, and 5-year survival was 73%, 36%, and 18%, respectively [38–40]. One should note that 44% of these patients had graft loss in the absence of tumor recurrence [39]. This may be explained by the lack of standardized immunosuppression protocols and operative expertise prior to 1995 [39].  Table 1 shows the results of published cohorts for LT in NRCLM.

The results of LT for NRCLM before 2000 were discouraging. Much of these results may be attributed to lack of standardized criteria for patient selection, basic technological resources, novice surgical expertise in LT, and the absence of standardized immunosuppression protocols. Indeed, in many initial experiences, the postoperative mortality after LT was high [28]. Furthermore, chemotherapy regimens for CRC in that era were not associated with good outcomes [43]. Due to a poor 5-year survival (<20%) and a high recurrence rate, the initial excitement of transplanting patients with NRCLM wined down and became a formal contraindication for LT worldwide.

## 4. Results of LT for Nonresectable CRC Liver Metastases after 2000

In 2006, the group from the Oslo University Hospital acquired ethical approval to conduct an open, prospective pilot study to assess the survival in NRCLM after LT (SECA Trial) [41]. Norway has a fortunate donor situation, with more donors than potential recipients, and the median waiting time for LT is less than 1 month [41]. The inclusion criteria for this trial were R0 primary colorectal resection; at least 6 weeks of one or more chemotherapy agents received for metastatic disease; nonresectable liver metastases; no extrahepatic disease; and ECOG performance status 0-1. All patients that qualified underwent an intraoperative staging laparotomy to examine the hepatic ligament lymph nodes before the transplant. If there was no disease in the frozen sections then the transplant was performed [41].

The study enrolled 25 patients of which 21 underwent deceased-donor LT. Four patients dropped out because they have developed extrahepatic disease. The median follow-up time was 27 (8–60) months and the 1-, 3-, and 5-year OS was 95%, 68%, and 60%, respectively. Recurrence occurred in 19 (90%) patients and 6 (29%) patients died of disseminated CRC after a median of 26 (6–41) months. Seven patients had pulmonary-only metastases and seven patients had CRC recurrence in the transplanted liver associated with other sites. No preoperative or adjuvant chemotherapy standardized protocol was administrated in this study and the posttransplant immunosuppressive protocol included sirolimus [41]. The main limitations of this study were the small sample and the relative short follow-up time (i.e., only 1 patient was evaluated 5 years after LT) [24]. Nevertheless, this study raised again the conundrum of transplanting patients with this NRCLM.

In a follow-up publication, the group from Oslo investigated the patterns of recurrence after LT for NRCLM [44]. The median time to recurrence was 6 months and all patients followed for longer than 11 months experienced recurrence. The lungs were the first single site of recurrence in 13/21 patients. Seven patients had lung-only metastasis and 3 underwent pulmonary resections. After a median follow-up of 27 months, all these 7 patients were alive. Interestingly, no patients had liver-only disease and in none the liver was the first recurrence site. In their protocol, patients with recurrent disease were always assessed for resection. Patients not eligible to salvage resection received locoregional therapies and/or palliative chemotherapy [44]. Despite the high recurrence rates, patients with pulmonary first-site metastases had a 5-year survival of 72% after recurrence was diagnosed. This fact made the authors conclude that pulmonary metastases after LT may be indolent and may not affect survival significantly [44]. In fact, a 5-year OS of ~45% has also been shown after multiple lung and liver resections of CRC LM [45, 46].

In 2005, the same group published a retrospective study comparing the SECA Trial Cohort to the NORDIC VII Trial Cohort [47]. The NORDIC VII Trial was a multicenter randomized 3-arm trial to assess the OS between fluorouracil/folinic acid and oxaliplatin (FLOX) when administered in bolus (Nordic FLOX) and FLOX combined to cetuximab and intermittent FLOX associated with cetuximab in patients with advanced CCR [48]. The 47 patients with liver-only metastases who did not undergo liver resection in the NORDIC VII Trial (and therefore were treated with chemotherapy only) were compared to the 21 patients that underwent LT in the SECA Trial [47]. The two groups were comparable except for the CEA levels (the SECA Trial cohort had a median of 15 *μ*g/L compared with 42 *μ*g/L in the NORDIC VI Trial). All patients in the NORDIC VII received first-line chemotherapy while 57% patients on the SECA trial received second and third lines of chemotherapy. The 5-year survival of the SECA Trial cohort was 56% and the 5-year survival of the 21 patients with the longest survival in the NORDIC VII Trial cohort was 19% (*P* = 0.01) [47]. Given that there is no randomized controlled trials comparing LT to standard chemotherapy in patients with liver-only NRCLM, this is the best available evidence comparing LT with standard of care chemotherapy. However, the evidence of this cohort study is still weak with several bias, and further comparisons are needed.

Recently, a European consortium has published a series of 12 patients that underwent LT for NRCLM [42]. Patients underwent LT after a median of 41 months following the primary CRC resection and 11 patients received chemotherapy before LT. Irinotecan and oxaliplatin were the most common protocols. Six patients had received biological (i.e., bevacizumab) agents prior to LT. LT was part of the planned treatment strategy in 6 patients whereas in 6 patients LT was not within the initial planned treatment strategy. The median follow-up was 26 months and the OS at 1-, 3-, and 5-year was 83%, 62% and 50%, respectively. Six patients had recurrence, mainly to the lungs (5 patients) [42]. The most salient finding of this study (compared to previous cohorts) is that 4 patients were alive without cancer recurrence after ~48 months. This study was not a prospective trial and has a very small sample. Moreover, this report did not include a standardized patient selection and intervention protocols. Due to this lack of standardization, these results must be interpreted with caution. Nevertheless, this is the first report to show that long-term cure can be achieved with LT in these patients and therefore the results are very encouraging.

The results of LT for NRCLM after the year 2000 were better than in the previous era, with a 5-year survival around 50%. This is likely related to changes in the selection criteria but probably more importantly to the development of effective chemotherapy regimens and the dramatic improvements in the perioperative care of LT recipients [49]. On the other hand, recurrence remains high. The patterns of recurrence in CRC have changed in the last few years [50]. Patients with initial NRCLM can be convert to resectable disease [51]. Multiple metastatic disease sites can be resected [52], and adjuvant chemotherapy can control tumor progression [10]. Certainly tumor recurrence impacts on patients survival; nevertheless, with these advances and control of mortality after recurrence, metastatic CRC is being described and sometimes considered as a chronic disease [49, 53, 54]. Definitively patients may have a long life expectancy with metastatic disease [44, 46].

## 5. Liver Graft Scarcity: Living-Donor Liver Transplantation as an Alternative

As a result of the tremendous success of LT for many indications, there has been an increment increase of the demand for grafts [55]. In the vast majority of LT centers, the number of patients waiting for a LT is greater than the number of available grafts, with the dramatic consequence of a high waiting list mortality [25]. The Oslo experience was made feasible by virtue of the fact that the authors practices in a region were the number of deceased donors which is higher than the numbers of recipients in the waiting list [41]. This is an unique situation and, unfortunately, most of the LT centers face a dramatic organ shortage. In this regard, the LT allocation criteria were thereby developed to distribute the organs to patients with the greatest likelihood of survival [56]. Accounting for the graft shortage, distributing deceased donors grafts to patients with advanced CRC does not seem appropriate (at this point) as most likely it will impact on other patients in desperate need of a graft.

Several strategies have been developed to increase the organ pool [25]. Donation after circulatory death and the use of marginal organs (i.e., older or steatotic grafts) are proven to be feasible [57–59]. However these are known to be more prone to enhanced ischemia reperfusion injury which translates as early graft dysfunction, primary graft nonfunction or severe biliary tract injury [60]. The split liver transplantation, in which an organ is divided to enable use by two recipients, is also an alternative to offer LT to more patients but has not been widely implemented [61]. Moreover, efforts have been made to improve the identification process of potential brain death donors to discuss organ donation with patients' relatives [62].

Living-donor liver transplantation (LDLT) may be an appealing alternative for selected patients with NRCLM. LDLT has become safer and more popular (mainly in Asian centers) with the refinement of surgical technic and the understanding of “small for size syndrome” [63]. There are two main advantages of LDLT. First, LDLT offers a source of liver grafts that does not impinge upon the deceased-donor organ pool [64]. Second, LDLT is performed in an “elective” basis. This may be specially interesting in the context of metastatic disease given that patients with NRCLM need to complete a preoperative treatment (e.g., neoadjuvant chemotherapy and restaging right before the LT). In this regard, our group is exploring the role of LDLT for the management of patients with NRCLM (NCT02864485) [65]. Patients deemed as having NRCLM will undergo at least 6 months of standard of care chemotherapy and thereafter a LDLT. The primary outcome is OS and the first results are planned to be published in 2020. Certainly the main concern when utilizing LD grafts is donor safety and therefore such approach needs to be conducted in high volume centers [66, 67].

## 6. Future Perspectives

There are several ongoing trials to further address the potential of LT for NRCLM. The Oslo University Hospital Group is recruiting patients for the SECA-II Trial (NCT01479608) [68]. The SECA-II Trial is an open label, randomized controlled trial to assess the OS between patients undergoing LT or liver resection. The study is planned to be complete in 2027. The same group established the RAPID (Resection and Partial Liver Segment 2/3 Transplantation with Delayed Total Hepatectomy) Trial (NCT02215889) [69]. The RAPID concept is to perform a left lateral segmentectomy and orthotopic transplantation of a left lateral segment graft. The total hepatectomy is delayed until the transplanted graft has reached sufficient volume [70]. The RAPID Trial will assess the safety and benefit of this technic in transplanted patients receiving second hepatectomy within 4 weeks of segment 2/3 implantation and OS. The study is planned to be completed in 2028. The TRANSMET Trial from France (NCT02597348) is recruiting patients to a randomized open label trial. Patients with NRCLM will be randomized to receive standard of care chemotherapy or LT plus chemotherapy [71]. The main outcome is 3- and 5-year disease-free survival/progression-free survival. The results are planned to be published in 2027.

If the ongoing phase II trials are able to prove that LT is safe and effective for the treatment of NRCLM, the need of a large randomized controlled trial may rise. This need will bring an ethical dilemma: would it be ethical to randomize patients to receive SOC if we have noncomparative studies showing better survival with LT? Probably not. Despite being the gold-standard in medical research, randomization is many times challenging in the field of transplantation [72]. Therefore, studies in the field of Transplant Oncology may just need to focus on comparability rather than randomization. Furthermore, the growing evidence of the benefit of LT for patients with NRCLM may push the transplant community to accept these patients for transplantation even before the results of the ongoing results trials are definitive (in 10 years' time). This has certainly been the case with LT for other indications such as HCC.

## 7. Conclusion

LT may offer a survival benefit to patients with NRCLM. However, it will remain controversial until high quality prospective studies can show significant survival improvement. Future studies should focus on patient selection criteria to achieve lower recurrence rates. The use of LDLT for NRCLM is promising and may impact the field positively by increasing the pool of grafts available without affecting other patients in the waiting list. In conclusion, the field of LT for NRCRC has changed substantially in the last years and, after adequate risk stratification and patient selection, LT may become clinically applicable and integrated into our management guidelines.

## Figures and Tables

**Table 1:** Published series on LT for NRCLM.

Study	Year	*N*	Overall survival			Recurrence
			1 year	3 years	5 years	
Prior to 2000						
Mühlbacher et al. [33]^¶^	1991	25	76%	32%	12%	64%
Penn [37]^*∗*^	1991	10	38% in 2-years		21%	70%
ELTR [39]	1995	58	73%	36%	18%	
After 2000						
Hagness et al. [41]	2013	21	95%	68%	60%	90%
Toso et al. [42]	2017	12	83%	62%	50%	50%

^¶^Survival data extracted from [35]. ^*∗*^Two patients were transplanted due to chemotherapy complications.
